# Vancomycin Dosing in Neutropenic Patients

**DOI:** 10.1371/journal.pone.0112008

**Published:** 2014-11-12

**Authors:** Michiel B. Haeseker, Sander Croes, Cees Neef, Cathrien A. Bruggeman, Leo M. L. Stolk, Annelies Verbon

**Affiliations:** 1 Department of Medical Microbiology, Maastricht University Medical Center, Maastricht, the Netherlands; 2 Care and Public Health Research Institute (CAPHRI), Maastricht, the Netherlands; 3 Department of Clinical Pharmacy and Toxicology, Maastricht University Medical Center, Maastricht, the Netherlands; 4 Department of Internal Medicine, Erasmus Medical Center, Rotterdam, the Netherlands; Northeastern University, United States of America

## Abstract

**Background:**

To compare vancomycin pharmacokinetic parameters in patients with and without neutropenia.

**Methods:**

Patients ≥18 years admitted on general wards were included. Routinely vancomycin trough and peak plasma concentrations were measured with a fluorescence polarization immunoassay. Pharmacokinetic parameters of individual patients were determined with maximum a posterior Bayesian estimation (MW Pharm 3.60). Neutropenia was defined as neutrophils <0.5×10^9^ cells/L.

**Principal Findings:**

A total of 171 patients were included. Patients with neutropenia (n = 56) had higher clearance of vancomycin (CLva), 67 (±26) mL/min, compared to patients without neutropenia (n = 115), CLva 50 (±22) mL/min (p<0.001). No significant difference was found in serum creatinine and vancomycin volume of distribution. Neutropenia was positively associated with CLva, independently of relevant co-variables (B: 12.122, 95%CI: 1.095 to 23.149, p = 0.031). On average patients with neutropenia needed 33% higher doses of vancomycin to attain adequate exposure, i.e. AUC_24_≥400 mg×h/L. Furthermore, 15 initially neutropenic patients in our study group received vancomycin for a second administration period. Ten patients received the second administration period during another neutropenic period and 5 patients during a non-neutropenic phase. All 5 patients with vancomycin during both neutropenic and non-neutropenic phase had higher CLva (91 (±26) mL/min) during the neutropenic period and lower CLva (45 (±10) mL/min) during the non-neutropenic phase (p = 0.009).

**Conclusion:**

This study shows that most patients with neutropenia have augmented CLva. In a small group of patients that received vancomycin during two episodes, the augmented CLva seems to be reversible in the non-neutropenic period. Our data indicate that it is important to increase the daily dose with one third in patients with neutropenia (from 15 mg/kg twice daily to 13 mg/kg three times daily). Frequent performance of therapeutic drug monitoring in patients with neutropenia may prevent both therapy failure due to low AUCs and overcomes toxicity due to high vancomycin trough concentrations during recovery from neutropenia.

## Introduction

Mortality from infections after cytostatic conditioning regimens in hematologic neutropenic patients requiring hematopoietic cell transplantation is high [1]. Bacterial infections are common during neutropenic phases and antibiotics, such as vancomycin, are often required [2]. In a recent surveillance study, Gram positive organisms are the most common cause of bacteremia in hematology patients, i.e. coagulase negative staphylococci (36%), followed by, Streptococci (11%), *S. aureus* (8%) and Enterococci (4%) [3]. Antibiotics should be started within 1 hour in patients with severe sepsis. However, adequate dosing of vancomycin can be difficult. Augmented clearance has been increasingly described in critically ill patients at the Intensive Care Unit (ICU) [4]–[6]. Changes in volume of distribution (Vd), changes in renal function and severe hypoalbuminemia are often present, influencing vancomycin plasma concentrations. Augmented clearance of vancomycin leads to lower vancomycin plasma concentrations, decreased 24-hour area under the curve (AUC_24_) and leads to diminished clinical outcome [6]. Augmented clearance of vancomycin in patients with hematological malignancies has been reported, but the augmented clearance was not associated with population specific covariables [7]. In another study low teicoplanin trough concentrations in neutropenic patients were reported, suggesting augmented clearance of teicoplanin in neutropenic patients [8]. In addition, elevated clearance of piperacillin and ceftazidime has also been noticed in patients with febrile neutropenia [9], [10]. The mechanism of augmented clearance of antibiotics is not completely understood and is poorly investigated in patients with hematologic malignancies or in patients with neutropenia. The aim of this study is to compare vancomycin pharmacokinetic parameters in patients with and without neutropenia at non-ICUs in a University Hospital.

## Methods

### Materials and Methods

#### Study group

In this observational study patients were prospectively followed. Patients older than 18 years treated with vancomycin intravenously (iv) and hospitalized at the Maastricht University Medical Center (MUMC), a 715 bed university hospital, were included from May 2011 until July 2013. Patients were excluded when admitted at the ICU or when insufficient data was collected. Vancomycin was started at the discretion of the attending physician, either empirically or as therapy for bacteria susceptible to vancomycin. Dose individualization was applied since an initial loading dose of 15 mg/kg was followed by dose adjustment based on therapeutic drug monitoring (TDM) and renal function. Demographic and clinical data, such as age, gender, weight, temperature, co-medication, length of hospital stay, time of administration of vancomycin and laboratory parameters, such as, serum creatinine (Jaffé method), and leucocytes were retrieved from the electronic patient file (SAP, the Netherlands). Neutropenia was defined as <0.5×10^9^ cells/L. Creatinine clearance (CLcr) was calculated with the Cockcroft and Gault formula [(140 - age in years) × weight in kg]/[serum creatinine in µmol × factor] using total bodyweight [11].

#### Ethics statement

This study was conducted according to the principles expressed in the Declaration of Helsinki. This study was registered at the Dutch Trial Register (NTR 1725). The Medical Ethical Committee of the Maastricht University Medical Center (MEC 08-4-063) approved this study and waived the necessity to obtain informed consent from participants because of the observational design. Electronic health records were anonymized prior to use.

#### Measurement of vancomycin

Vancomycin plasma concentrations were measured as standard clinical care with a fluorescence polarization immune assay using of Cobas Integra 800 system (Roche Diagnostics). The calibration curve ranged from 2.0 to 80 mg/L. The accuracy and coefficients of variation (CV) of the controls (6.9, 17.7 and 31.0 mg/L) were within 90%–110% and <3.3%, respectively. Patients with at least two plasma samples available, drawn in such a manner to ensure calculations of vancomycin clearance (CLva) were included. Blood samples were collected at least one hour after the end of infusion and trough levels were obtained just before the next dose.

#### PK-analysis

Pharmacokinetic parameters (CLva, Vd) of vancomycin in individual patients were calculated with maximum a posterior (MAP) Bayesian estimation computer program (MW/Pharm 3.60, Mediware, the Netherlands). Bayesian priors from a two compartment open pharmacokinetic model based on previous studies, were applied: V1 0.21±0.04 L/kg, k_elm_ 0.0143±0.0029 h^−1^, k_elr_ = k_slope_ × CLcr (mL/min), 0.00327±0.00109 h^−1^/mL/min, k_12_ 1.12±0.28 h^−1^, and k_21_ 0.48±0.12 h^−1^ [12], [13], where V1 is volume of distribution central compartment (L/kg); k_elm_, metabolic elimination rate constant (h^−1^); k_slope_, renal elimination rate constant (h^−1^/mL/min); k_elr_, renal elimination rate constant (h^−1^); k_12_ (h^−1^), rate constant from the 1^st^ to the 2^nd^ compartment; and k_21_(h^−1^), vice versa. The elimination rate constant k_el_  =  k_elm_ + k_elr_  =  k_elm_ + (k_slope_ × CLcr) [14]. With MAP Bayesian estimation all patient characteristics and measured vancomycin concentrations are fitted on an existing population model. With at least two concentrations per patient individual pharmacokinetic parameters can be adequately derived with MAP Bayesian estimation [15], [16]. With these individual pharmacokinetic parameters, dosing simulations were made to adjust the dose individually; this MAP Bayesian estimation is a standard procedure in institutes which provide TDM service.

The AUC_24_ in steady-state was calculated with the formula: 24-hour dose/CLva.

#### Analysis of patients with and without neutropenia

Pharmacokinetic, clinical and demographic parameters were compared in patients with and without neutropenia in all patients and in patients with hematological malignancies. Furthermore, pharmacokinetic parameters of two vancomycin administration periods within the same patients were compared. Both patients with two vancomycin administrations during two different neutropenia periods and patients with two vancomycin administrations during one neutropenia period and one period without neutropenia were compared.

#### Statistical analysis

Normal distribution was evaluated for metric variables by means of the Shapiro-Wilk test and presented as mean (±SD). If not, median and ranges were given. Categorical variables are presented as frequencies and percentages. Metric and categorical variables were evaluated between patients with and without neutropenia using the Student t-test or non-parametric test (Kruskal Wallis), respectively.

First, the influence of co-variables on CLva was determined in univariable (Pearson) analysis. Subsequently, only the significant co-variables in the univariable analyses were included in the multivariable analysis, after checking the assumptions. The Enter method was used in the multivariable linear regression. CLcr is estimated with the C&G formula which includes serum creatinine, age, weight and gender [11]. To avoid multicollinearity, serum creatinine, age, weight and gender were left out of the multivariable model. Data analysis was done with IBM SPSS-pc version 20.0. A p-value of <0.05 was considered to be statistically significant.

## Results

### Study group

The mean age was 59 (±14) years and 61% were male. Patients were admitted on different general wards; hematology ward (40%, 68/171), surgery ward (19%, 32/171), internal ward (11%, 19/171), neurosurgery ward (11%, 18/171), orthopedic ward (10%, 17/171), cardiac (9%, 15/171) and eye ward (1%, 2/171). The majority of patients had sepsis (46%, 79/171), implant infection (16%, 27/171) or abdominal infection (15%, 25/171). A total of 171 patients with a mean (±SD) of 6 (±3) vancomycin plasma concentrations were included.

### Pharmacokinetics analysis

The mean dose (±SD) of vancomycin per 24 hours was 1683 (±759) mg, with a mean Vd of 58 (±30) L and AUC_24_ of 502 (±97) mg×h/L. The mean (±SD) trough concentration in steady state (SS) was 13 (±4) mg/L, CmeanSS was 21 (±4) mg/L, peak concentration in SS was 49 (±14) mg/L, CLva was 56 (±25) mL/min and serum creatinine was 89 (±68) µmol/L.

### Analysis of patients with and without neutropenia

**Figure 1:**
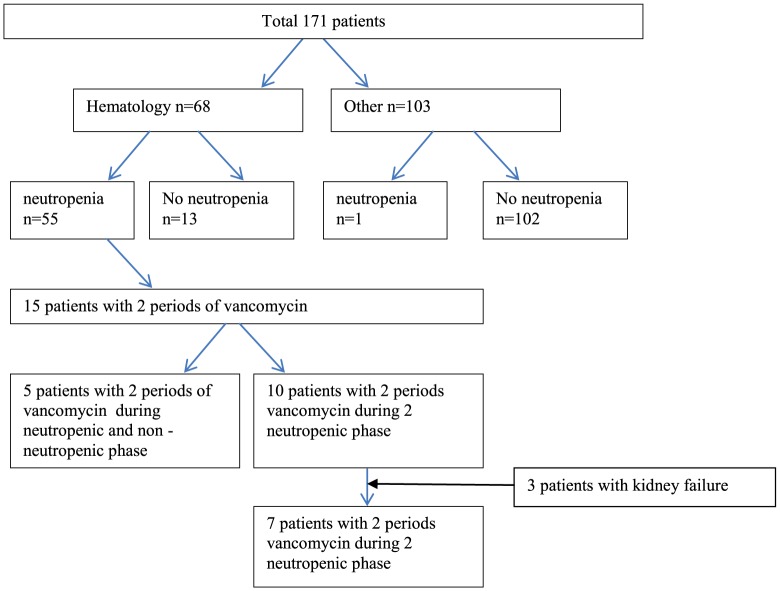
Flow of the 171 included patients with regard to hematology, neutropenia and two vancomycin administration periods.

**Figure 2:**
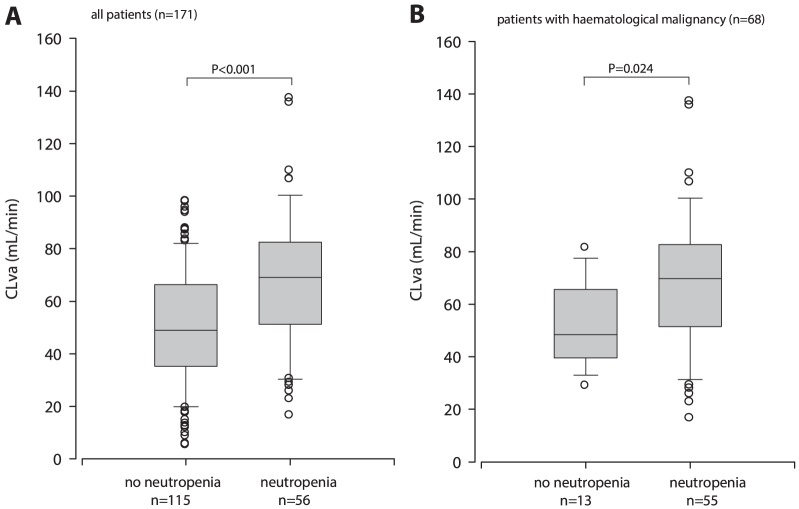
Boxplot for vancomycin clearance (CLva) in patients with and without neutropenia in all patients (A) and in patients with haematological malignancy (B). Lower and higher boundary of the box indicates 25^th^ and 75^th^ percentile, respectively, the line within the box marks the median, the whiskers above and below the box indicate the 90^th^ and 10^th^ percentiles and the open circles indicate outside the 90^th^ and 10^th^ percentiles.

Sixty eight patients had a hematological malignancy and 56 patients were neutropenic, Figure 1. Neutropenic patients (n = 56) had higher CLva, 67 (±26) mL/min, compared to non-neutropenic patients (n = 115), CLva 50 (±22) mL/min (p<0.001). No significant difference in serum creatinine and Vd was found, Table 1 and Figure 2. Forty eight percent (27/56) of the neutropenic patients had CLva >70 mL/min, compared to 21% (24/115) without neutropenia. Of the 68 patients with a hematological malignancy, 55 patients were neutropenic and 13 were not neutropenic. Within the hematologic malignancy patients, neutropenic patients had higher CLva, than non-neutropenic patients, Table 1 and Figure 2. Physicians used TDM and adjusted vancomycin dosing as shown by the mean dose of vancomycin in patients with neutropenia of 2017 (±720) mg compared to 1521 (±727) mg in patients without neutropenia, p<0.001. On average, among patients with neutropenia the daily vancomycin dose was 33% (500 mg/day) higher to achieve the same AUC_24_ (Table 1). Patients with sepsis (n = 79) had higher CLva and were younger than patients without sepsis (n = 92). Vd and CLcr were not different, Table 2. Neutropenic patients with sepsis (n = 47) seemed to have slightly higher CLva of 69 (±27) mL/min than neutropenic patients without sepsis (n = 9) CLva 60 (±22) mL/min, p = 0.269. Both neutropenic patients with sepsis and without sepsis had higher CLva than non-neutropenic patients.

**Table 1:** Mean (±SD) for Age, CLcr, CLva, Vd, Dose 24 h and AUC of patients with and without neutropenia in all patients (A) and in patients with haematological malignancy (B).

*A] All patients (n = 171)*								
Neutro-penia	N	Age year	CLcr mL/min	CLva mL/min	Creatinineµmol/L	Vd L	Dose 24 h mg	AUC mg*24 h/L
No	115	61(±14)	107 (±78)	50(±22)	95(±67)	56(±29)	1521(±727)	499(±102)
Yes	56	55(±13)	113 (±57)	67(±26)	80(±31)	62(±32)	2017(±719)	507(±87)
p		0.01	0.142	<0.001	0.873	0.304	<0.001	0.259
*B] Patients with haematological malignancy (n = 68)*								
Neutro-penia	N	Age Year	CLcr mL/min	CLva mL/min	Creatinineµmol/L	Vd L	Dose 24 h mg	AUC mg ×24 h/L
No	13	57(±11)	111(±58)	53(±16)	96(±59)	59(±18)	1604(±646)	502(±102)
Yes	55	55(±14)	114(±57)	68(±26)	79(±29)	62(±32)	2040(±705)	509(±87)
p		0.839	0.714	0.024	0.779	0.691	0.028	0.697

CLva: vancomycin clearance.

CLcr: creatinine clearance calculated from serum creatinine with Cockcroft and Gault [11].

Vd: volume of distribution.

AUC: 24 hour area under the curve.

**Table 2:** Mean (±SD) for Age, CLcr, CLva, Vd, Creatinine for patients with sepsis and without sepsis.

	N	Age years	CLva mL/min	Vd L	CLcr mL/min	Creatinine µmol/L
Sepsis	79	56 (±13)	60 (±27)	57 (±26)	108 (±56)	84 (±38)
No sepsis	92	61 (±14)	52 (±23)	58 (±33)	110 (±83)	96 (±71)
p		0.017	0.048	0.639	0.535	0.894

CLva: vancomycin clearance.

CLcr: creatinine clearance calculated from serum creatinine with Cockcroft and Gault [11].

Vd: volume of distribution.

**Figure 3:**
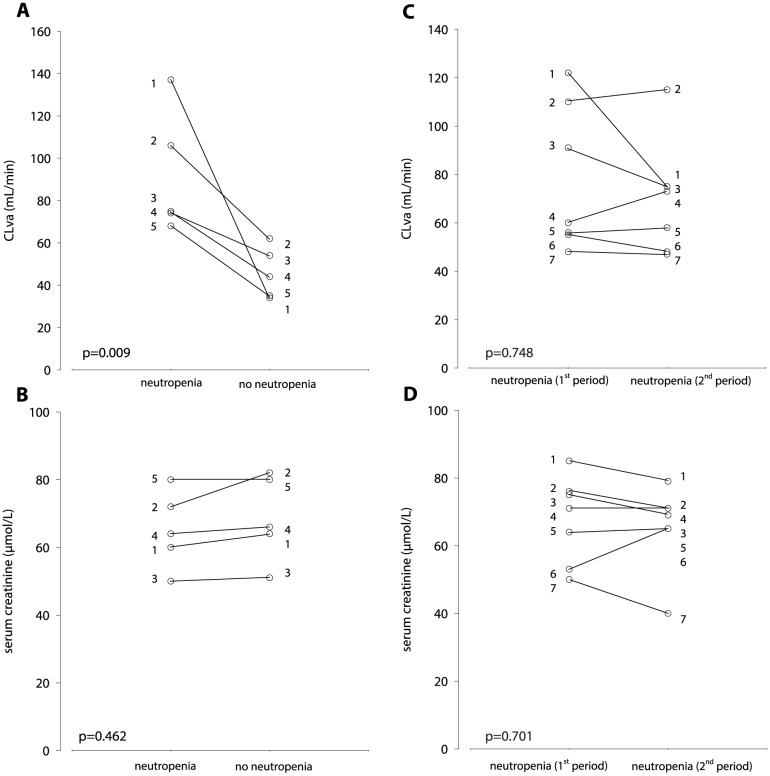
A. Vancomycin clearance (CLva) and B. serum creatinine of 5 patients (number 1–5) during both a neutropenic and a non-neutropenic phase and C. CLva and D. serum creatinine of 7 patients (number 1–7) during two neutropenic phases.

Of the 171 patients, 15 neutropenic patients received a second period of vancomycin, of which 5 patients received vancomycin during both an neutropenic and non neutropenic period. Ten patients received two vancomycin episodes during neutropenic periods. However, 3 patients developed kidney failure and were taken out. Leaving 7 patients with two vancomycin periods during neutropenia, Figure 1. Therefore, the data of 7 patients with two neutropenic periods and 5 patients with both a neutropenic and non-neutropenic period could be compared. The median (range) of time between the two vancomycin administrations was 30 (20–108) days for these 7 patients and 21 (14–136) days for the 5 patients with both a neutropenic and non neutropenic period. For the 7 patients with vancomycin administrations in two neutropenic periods, the CLva remained similar: 77 (±30) to 70 (±23) mL/min (p = 0.748), as did the serum creatinine 68 (±13) to 66 (±11) µmol/L (p = 0.701) and CLcr 120 (±41) to 117 (±35) mL/min (p = 0.848). The 5 patients with vancomycin administrations in both a neutropenic and non-neutropenic period had a statistically significantly higher CLva, 91 (±26) mL/min, during the neutropenic phase compared to CLva, 45 (±10) mL/min during the non-neutropenic phase (p = 0.009). Serum creatinine, 65 (±10) and 69 (±11) µmol/L (p = 0.462) and CLcr, 141 (±70) and 113 (±48) mL/min during the neutropenic and non-neutropenic periods, respectively, were not significantly different (p = 0.402), Figure 3 and neither was the Vd was 74 (±24) L during neutropenic and 51 (±10) L during non-neutropenic phase (p = 0.175).

CLcr, neutropenia, hematologic malignancy and sepsis were correlated with CLva in the univariable analysis, Table 3. In the multivariable analysis, CLva was positively associated with CLcr (B: 0.205, 95%CI: 0.164–0.245, p<0.001) and neutropenia (B: 12.122, 95%CI: 1.095 to 23.149, p = 0.031), Table 3.

**Table 3:** Univariable and multivariable correlation coefficients between CLva and predictors used in this study.

	Univariable^a^		Multivariable^b^			
	R	P-value	B	95% confidence interval for B		p-value
				Lower bound	Upper bound	
CLcr	0.599	<0.001	0.205	0.164	0.245	<0.001
Neutropenia	0.322	<0.001	12.122	1.095	23.149	0.031
Hematologic malignancy	0.300	<0.001	3.582	−8.404	15.569	0.556
Sepsis	0.170	0.027	0.427	−7.236	8.090	0.913
Vd	0.008	0.915	-	-	-	-

CLva: vancomycin clearance.

CLcr: creatinine clearance.

Vd: volume of distribution.

a Pearson correlation was performed as the univariable analysis.

b Only co-variates that were significantly correlated with CLva in the univariable analysis (P<0.05) were included in the multivariable analysis.

## Discussion

Our study shows that higher doses of vancomycin are needed during neutropenic periods to achieve vancomycin target AUC_24_ and target trough concentrations. The augmented clearance of vancomycin in neutropenic patients seems reversible. Augmented clearance of vancomycin cannot be predicted with the estimated CLcr, as serum creatinine and estimated CLcr in our study are not significantly different in neutropenic and non-neutropenic patients. Moreover, the estimated CLcr is not reliable above 125 µmol/L and shows a poor agreement with measured CLcr in urine in critically ill patients displaying augmented clearance of creatinine [17], [18]. Our Bayesian calculated CLva is in line with the population estimated CLva in patients with hematological malignancies in the simulations by Buelga *et al.* [7]. However, our routine patient care observations demonstrate that augmented clearance is associated with neutropenia rather than hematological malignancy and sepsis. In the multivariable analysis neutropenia (yes/no) was positively associated with CLva, independently of the other co-variables. Although, our group of patients that received a second administration of vancomycin is small, the augmented clearance of vancomycin seems to be temporarily and reversible, as the CLva returned to normal during the non-neutropenic phase. The mechanism of augmented clearance is not completely clarified; most likely more than one factor is involved in developing augmented clearance. Young age, increased blood flow to the kidneys, genetic factors and other medication has been proposed to influence the CLva [5], [6]. Neutropenia might be added to this list. Most likely augmented clearance also influences other renally cleared antibiotics [9], [10]. Therefore, TDM of these antibiotics or/and at least one 24-hour creatinine measurement in urine to determine the most accurate CLcr at the ICU is recommended [5], [19], [20]. Our data suggest that this recommendation may be extended to neutropenic patients.

Our study has a couple of limitations. Firstly, our study is a real-life observational study and we assumed the TDM protocol was strictly followed by clinicians, especially the timing of peak concentrations. Secondly, our group of patients with a second vancomycin administration was rather small to prove the demonstrated tendency of reversibility of elevated CLva, at the moment when patients are recovering from neutropenia. Further research is needed to fully understand the complex pharmacokinetics of vancomycin and other antibiotics in patients with neutropenia. A prospective study may elucidate which other factors are involved in augmented CLva, but such a study would need a multicenter design and inclusion of many patients. Until, we fully understand augmented clearance, we suggest to increase the initial daily dose of vancomycin with 33% (13 mg/kg three times daily) in patients with neutropenia and to perform TDM after the first vancomycin dose in patients to prevent low plasma concentrations of vancomycin and consequently reduced efficacy. When patients are recovering from neutropenia, TDM is again necessary to adjust the vancomycin dose to prevent toxicity due to high vancomycin exposure.

## References

[1] Scott BL, Park JY, Deeg HJ, Marr KA, Boeckh M, et al (2008) Pretransplant neutropenia is associated with poor-risk cytogenetic features and increased infection-related mortality in patients with myelodysplastic syndromes. Biol Blood Marrow Transplant 14: 799–806. 18541200 10.1016/j.bbmt.2008.04.011 PMC2587376

[2] Sepkowitz KA (2002) Antibiotic prophylaxis in patients receiving hematopoietic stem cell transplant. Bone Marrow Transplant 29: 367–371. 11919724 10.1038/sj.bmt.1703366

[3] Schelenz S, Nwaka D, Hunter PR (2013) Longitudinal surveillance of bacteraemia in haematology and oncology patients at a UK cancer centre and the impact of ciprofloxacin use on antimicrobial resistance. J Antimicrob Chemother 68: 1431–1438. 23396855 10.1093/jac/dkt002

[4] Revilla N, Martin-Suarez A, Perez MP, Gonzalez FM, Fernandez de Gatta Mdel M (2010) Vancomycin dosing assessment in intensive care unit patients based on a population pharmacokinetic/pharmacodynamic simulation. Br J Clin Pharmacol 70: 201–212. 20653673 10.1111/j.1365-2125.2010.03679.x PMC2911550

[5] Udy AA, Roberts JA, Boots RJ, Paterson DL, Lipman J (2010) Augmented renal clearance: implications for antibacterial dosing in the critically ill. Clin Pharmacokinet 49: 1–16. 20000886 10.2165/11318140-000000000-00000

[6] Claus BO, Hoste EA, Colpaert K, Robays H, Decruyenaere J, et al (2013) Augmented renal clearance is a common finding with worse clinical outcome in critically ill patients receiving antimicrobial therapy. J Crit Care 28: 695–700. 23683557 10.1016/j.jcrc.2013.03.003

[7] Buelga DS, del Mar Fernandez de Gatta M, Herrera EV, Dominguez-Gil A, Garcia MJ (2005) Population pharmacokinetic analysis of vancomycin in patients with hematological malignancies. Antimicrob Agents Chemother 49: 4934–4941. 16304155 10.1128/AAC.49.12.4934-4941.2005 PMC1315926

[8] Pea F, Viale P, Candoni A, Pavan F, Pagani L, et al (2004) Teicoplanin in patients with acute leukaemia and febrile neutropenia: a special population benefiting from higher dosages. Clin Pharmacokinet 43: 405–415. 15086277 10.2165/00003088-200443060-00004

[9] Pea F, Viale P, Damiani D, Pavan F, Cristini F, et al (2005) Ceftazidime in acute myeloid leukemia patients with febrile neutropenia: helpfulness of continuous intravenous infusion in maximizing pharmacodynamic exposure. Antimicrob Agents Chemother 49: 3550–3553. 16048982 10.1128/AAC.49.8.3550-3553.2005 PMC1196227

[10] Sime FB, Roberts MS, Warner MS, Hahn U, Robertson TA, et al. (2014) Altered pharmacokinetics of piperacillin in febrile neutropenic patients with haematological malignancy. Antimicrob Agents Chemother. 10.1128/AAC.02340-14 PMC4068433 24687508

[11] Cockcroft DW, Gault MH (1976) Prediction of creatinine clearance from serum creatinine. Nephron 16: 31–41. 1244564 10.1159/000180580

[12] Pryka RD, Rodvold KA, Garrison M, Rotschafer JC (1989) Individualizing vancomycin dosage regimens: one- versus two-compartment Bayesian models. Ther Drug Monit 11: 450–454. 2741194

[13] Rodvold KA, Pryka RD, Garrison M, Rotschafer JC (1989) Evaluation of a two-compartment Bayesian forecasting program for predicting vancomycin concentrations. Ther Drug Monit 11: 269–275. 2728085 10.1097/00007691-198905000-00009

[14] Manual MP. Available: http://www.mwpharm.nl/downloads/documentation/UK-315-VOL3.PD.

[15] van der Meer AF, Marcus MA, Touw DJ, Proost JH, Neef C (2011) Optimal sampling strategy development methodology using maximum a posteriori Bayesian estimation. Ther Drug Monit 33: 133–146. 21383653 10.1097/FTD.0b013e31820f40f8

[16] Proost JH, Meijer DK (1992) MW/Pharm, an integrated software package for drug dosage regimen calculation and therapeutic drug monitoring. Comput Biol Med 22: 155–163. 1617949 10.1016/0010-4825(92)90011-b

[17] Grootaert V, Willems L, Debaveye Y, Meyfroidt G, Spriet I (2012) Augmented renal clearance in the critically ill: how to assess kidney function. Ann Pharmacother 46: 952–959. 22693271 10.1345/aph.1Q708

[18] Hoste EA, Damen J, Vanholder RC, Lameire NH, Delanghe JR, et al (2005) Assessment of renal function in recently admitted critically ill patients with normal serum creatinine. Nephrol Dial Transplant 20: 747–753. 15701668 10.1093/ndt/gfh707

[19] Udy AA, Putt MT, Shanmugathasan S, Roberts JA, Lipman J (2010) Augmented renal clearance in the Intensive Care Unit: an illustrative case series. Int J Antimicrob Agents 35: 606–608. 20307958 10.1016/j.ijantimicag.2010.02.013

[20] Troger U, Drust A, Martens-Lobenhoffer J, Tanev I, Braun-Dullaeus RC, et al (2012) Decreased meropenem levels in Intensive Care Unit patients with augmented renal clearance: benefit of therapeutic drug monitoring. Int J Antimicrob Agents 40: 370–372. 22795654 10.1016/j.ijantimicag.2012.05.010

